# Supplementary material for: Tubeimosides are pan-coronavirus and filovirus inhibitors that can block their fusion protein binding to Niemann-Pick C1
Source: Nat Commun. 2024 Jan 2;15:162. doi: 10.1038/s41467-023-44504-4 (PMC10762260; doi:10.1038/s41467-023-44504-4)
Supplement: Supplementary file 1 — Supplementary Information [file 41467_2023_44504_MOESM1_ESM.pdf]

Supplemental Figures

Plasmids	F3-1	F3-2	F3-3	F3-4	F3-5
HIV-ΔEnv-Luc	+	+	+	+	+
EBOV-GP-wt	+				
EBOV-GP0-ΔMLD		+			
pCAGGS-Flag-SARS2-D19-S			+		
pCAGGS-Flag-SARS1-D19-S				+	
pCAGGS-Flag-MERS-D16-S					+

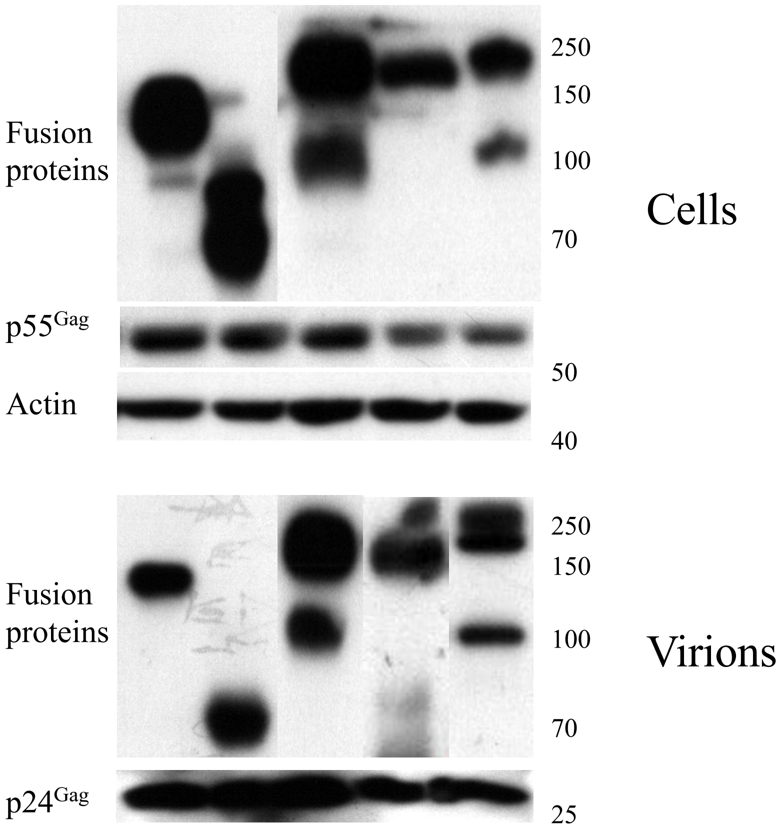

Fig. S1. HIV-1 psedovirions were produced from HEK293T cells by transfection with indicated vectors and virions were purified by ultracentrifugation. Viral protein expression was determined by WB. Experiments were repeated 3 times independently, and representative results are shown.

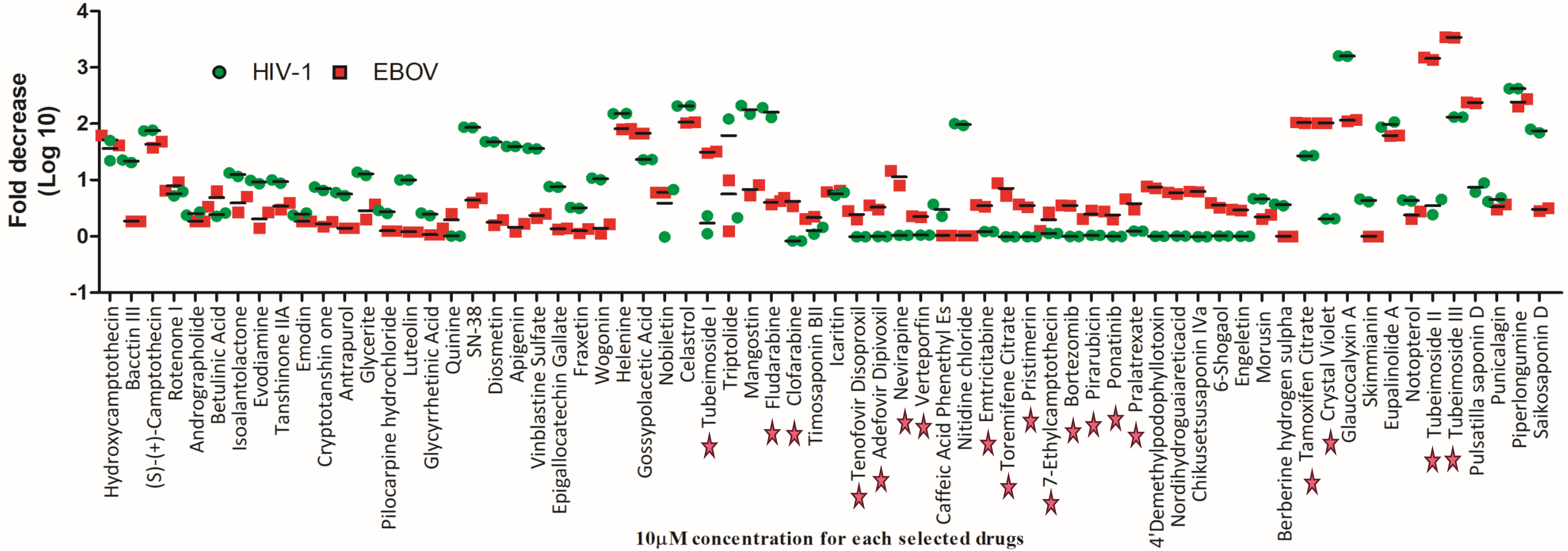

Fig. S2. A library containing 974 plant-sourced small compounds was screened at 10 μM for their antiviral activity to HIV-1 luciferase (Luc) reporter pseudovirions expressing Ebola virus glycoprotein (EBOV-GP) or HIV-1 envelope glycoprotein (HIV-1 Env). The screening for anti-EBOV (red) was conducted in Vero-E6 cells, and the screening for anti-HIV-1 was done in TZM-bI cells. Results shown are from one representative screening from these experiments.

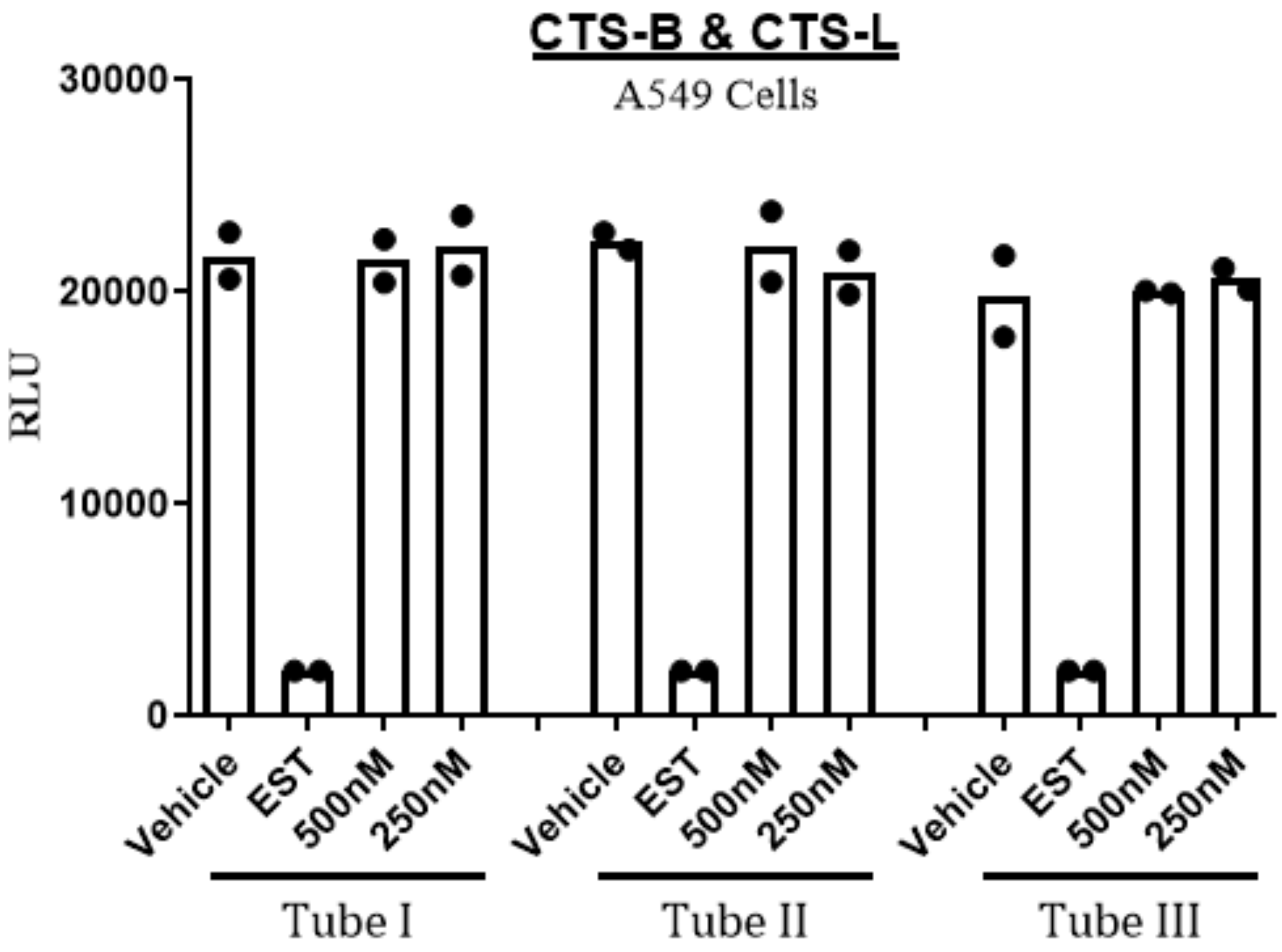

Fig. S3. Mixed Cathepsin B and L substrates were incubated with cell lysate from A549 cells treated with Tubs I, II, and III, and their activity was determined. E-64-d ethyl ester (EST) was used at 10 μM as a positive control.

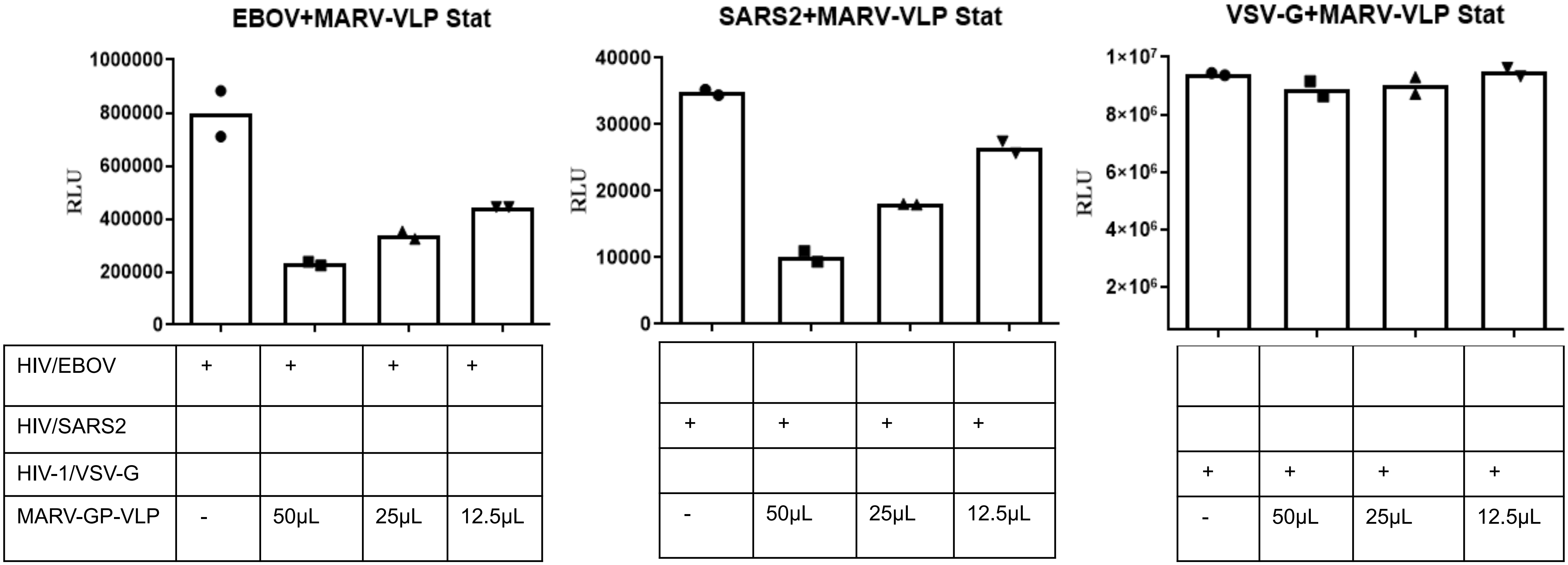

Fig. S4. Huh-7 or Huh-7-A-T cells were treated with EBOV-VLPs expressing MARV-GP and infected with HIV-1 Luc-pseudovirions expressing EBOV-GP, SARS2-S, or VSV-G. Viral infection was determined by measuring intracellular luciferase activity (RLU).

Knockout of *NPC1* in CHO cells by CRISPR/Cas9

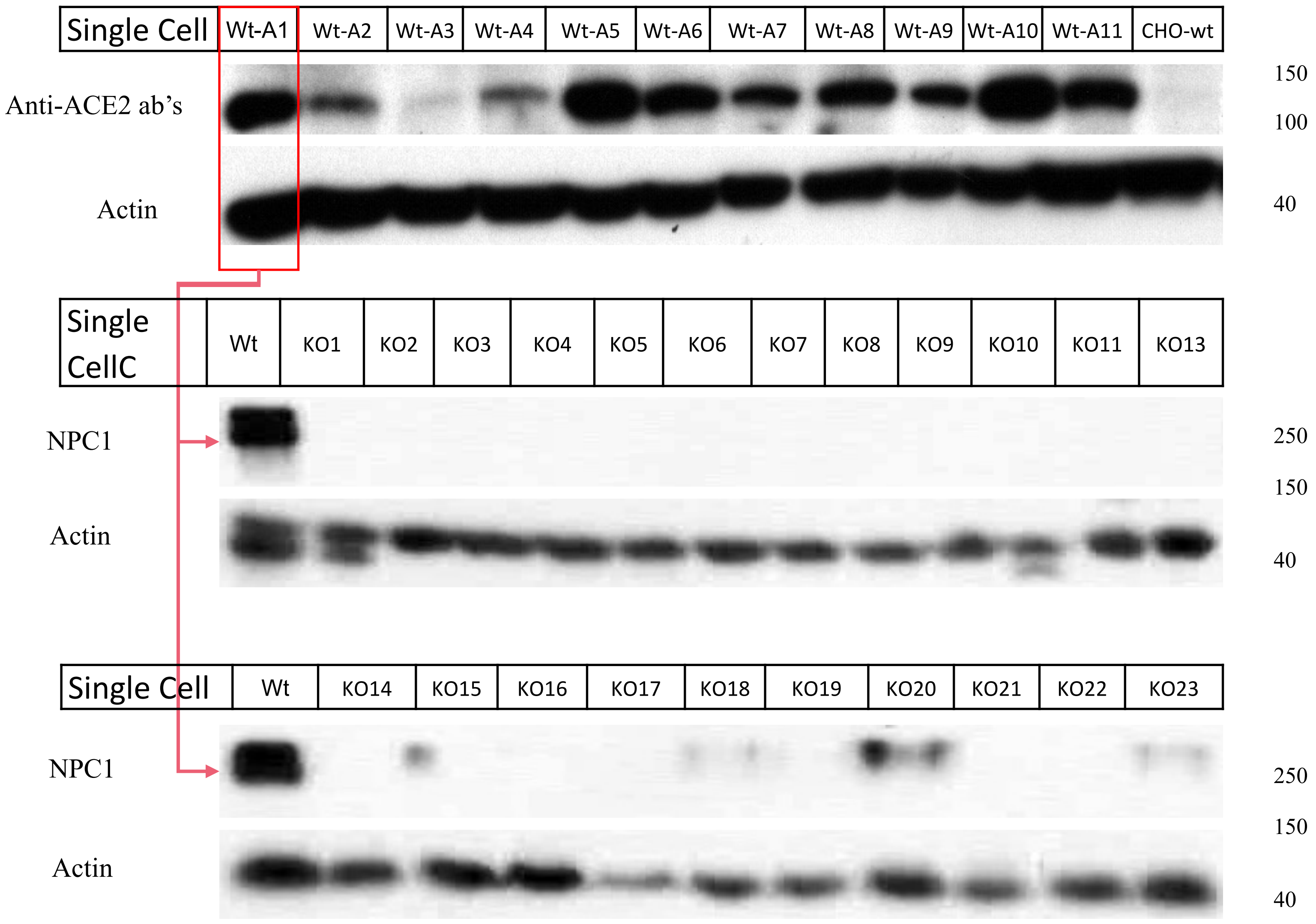

Fig. S5. CHO cells were transduced with a lentiviral vector expressing human ACE2 and stable clones were obtained after selection with blasticidin. CHO (WT) cells and CHO expressing ectopic ACE2 (Wt-A1) were then transduced with another lentiviral vector expressing CRISPR/Cas9 and *NPC1* sgRNAs and stable knockout (KO) clones were obtained after selection with puromycin. Results shown are western blots for selection of these cell clones. Experiments were repeated 3 times independently, and representative results are shown.

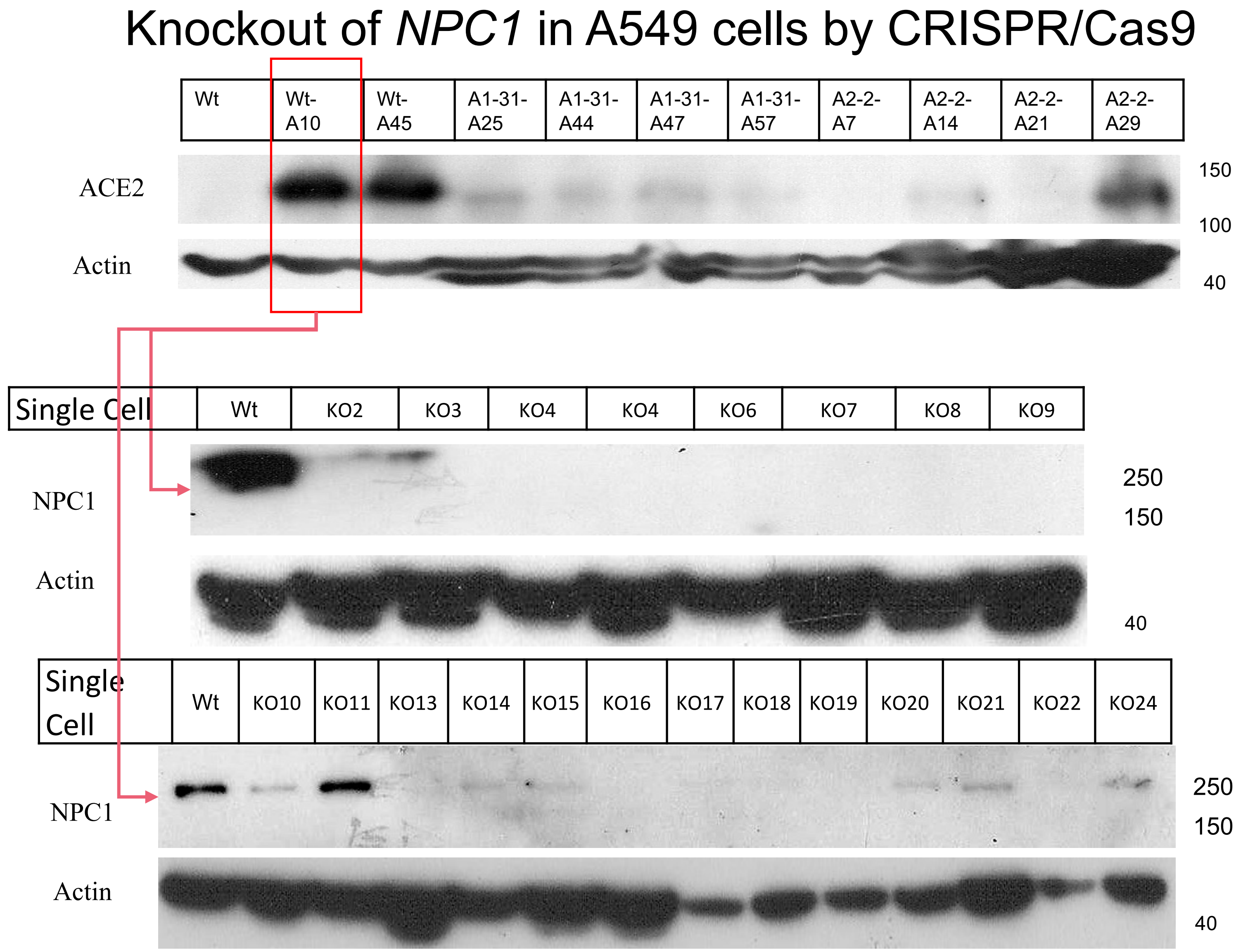

Fig. S6. A549 cells were transduced with a lentiviral vector expressing human ACE2 and stable clones were obtained after selection with blasticidin. A549 (WT) cells and A549 expressing ectopic ACE2 (Wt-A10) were then transduced with another lentiviral vector expressing CRISPR/Cas9 and *NPC1* sgRNAs and stable knockout (KO) clones were obtained after selection with puromycin. Results shown are western blots for the selection of these cell clones. Experiments were repeated 3 times independently, and representative results are shown.

Knockout of *NPC1* in Caco2 cells by CRISPR/Cas9

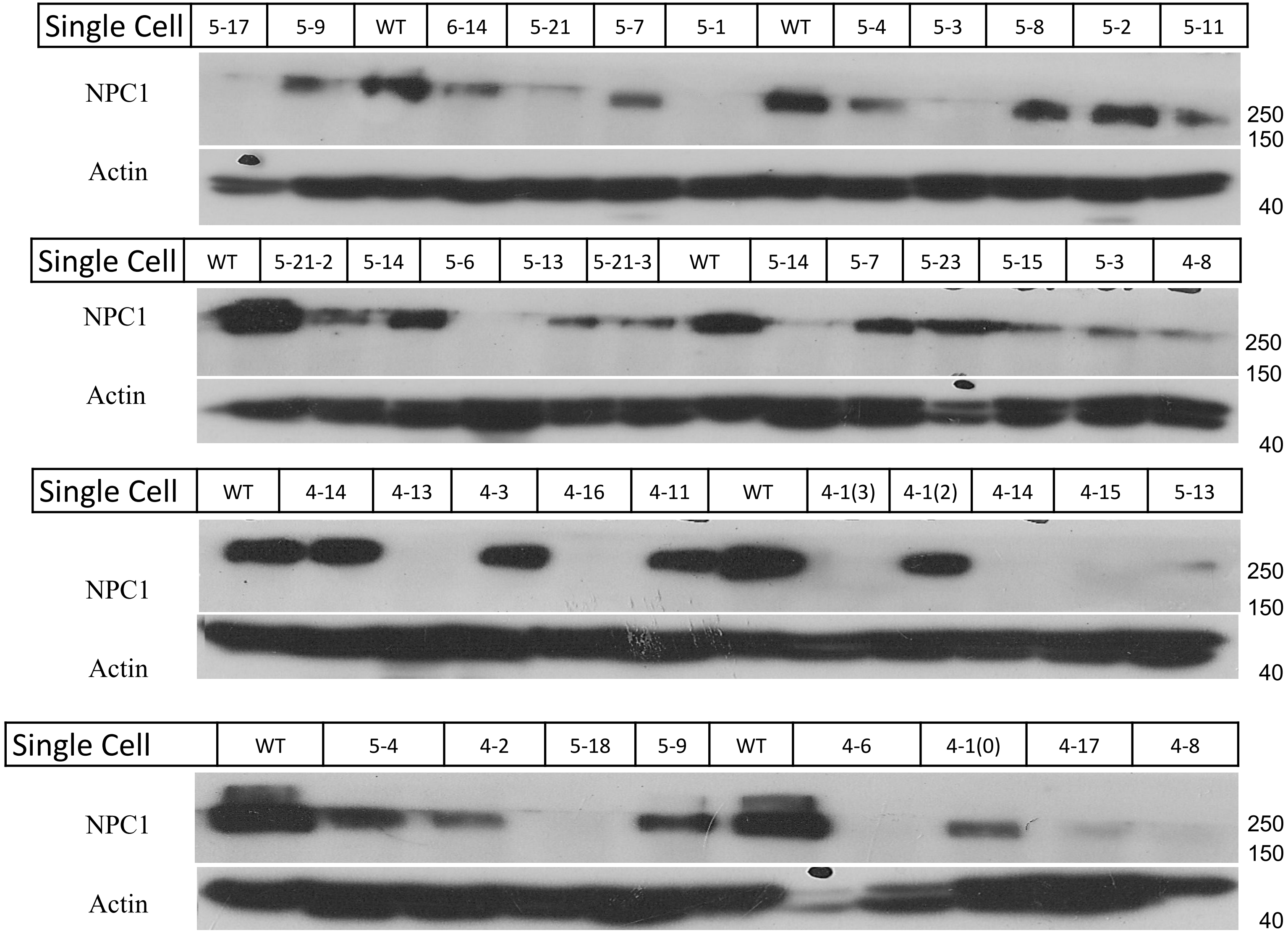

Fig. S7. Caco2 cells were transduced with a lentiviral vector expressing CRISPR/Cas9 and *NPC1* sgRNAs and stable knockout (KO) clones were obtained after selection with puromycin. Results shown are western blots for selection of these cell clones. Experiments were repeated 3 times independently, and representative results are shown.

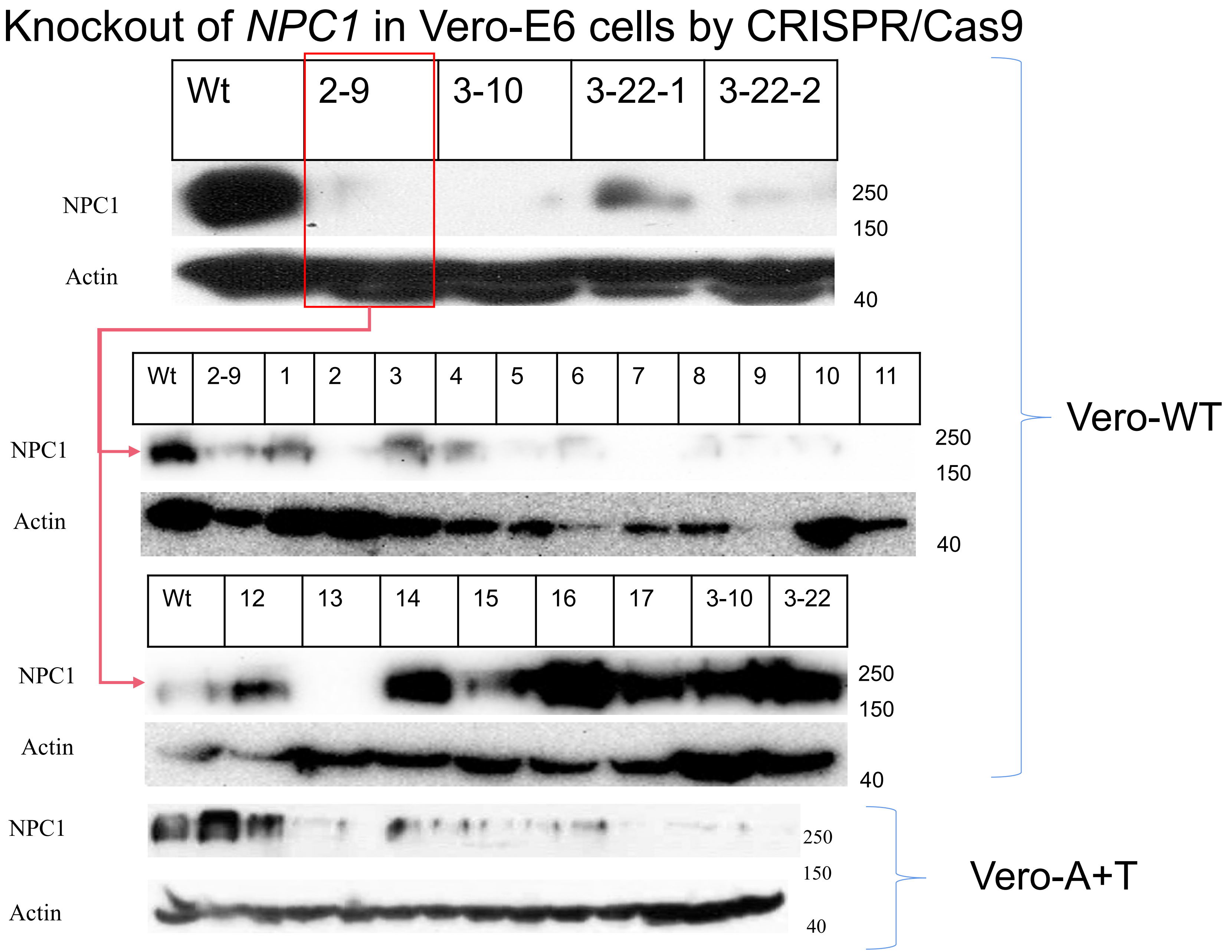

Fig. S8. Vero-E6 cells were transduced with lentiviral vectors expressing human ACE2 (A) or TMPRSS2 (T), and stable clones were obtained after selection with blasticidin. Vero-WT cells and Vero-WT-A+T cells were then transduced with another lentiviral vector expressing CRISPR/Cas9 and *NPC1* sgRNAs and stable knockout (KO) clones were obtained after selection with puromycin. Results shown are western blots for selection of these cell clones. Experiments were repeated 3 times independently, and representative results are shown.

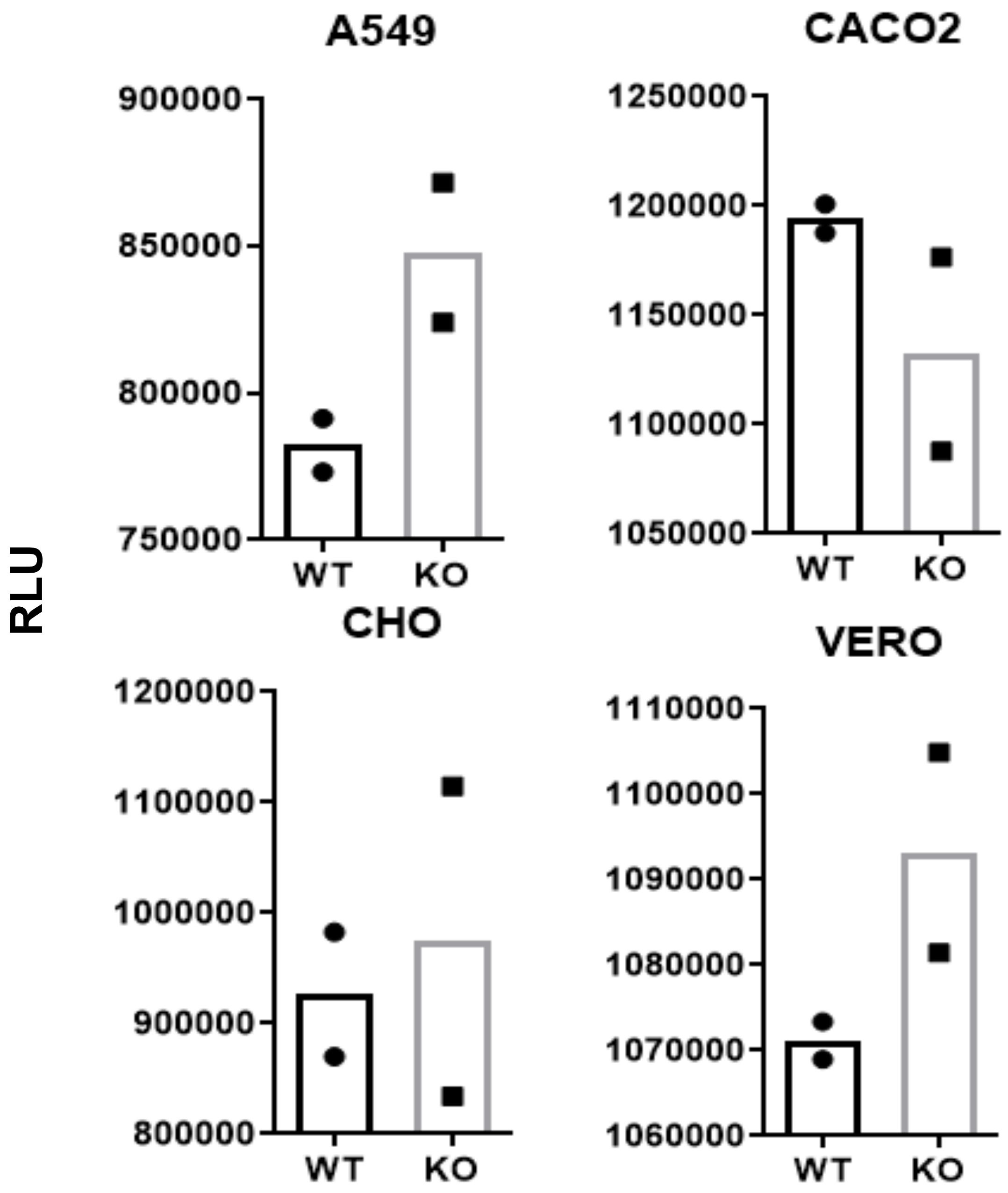

Fig. S9. The growth of indicated cells and their NPC1-KO cells were measured using CellTiter-Glo® Luminescent Cell Viability Assay kit (Promega).

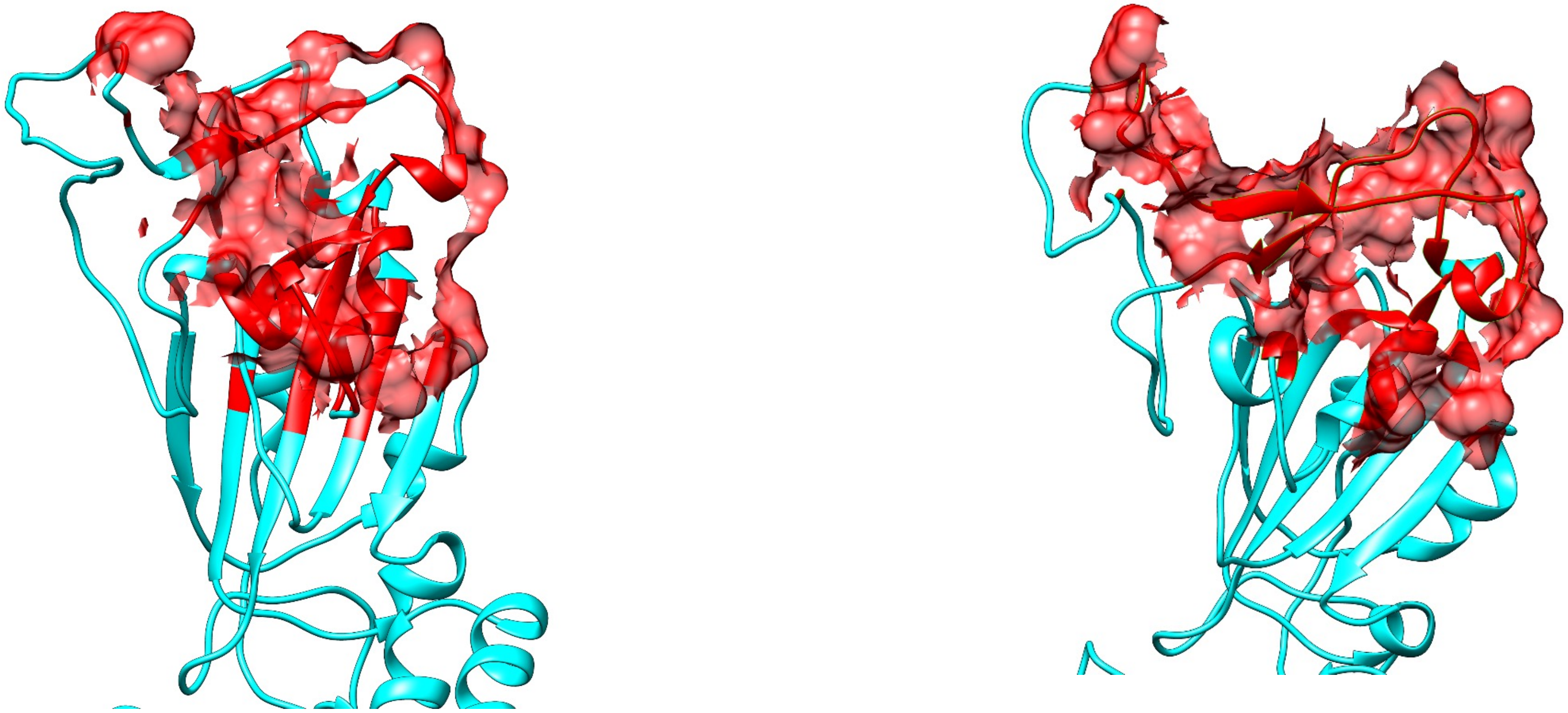

Fig. S10. Local view of the binding interface of SARS2-S (**left**) and Omicron-S (**right**) are presented.. The SARS2-S and Omicron-S structure are shown in cyan, and their binding interfaces, predicted to directly interact with NPC1-C, are highlighted in red as a surface.

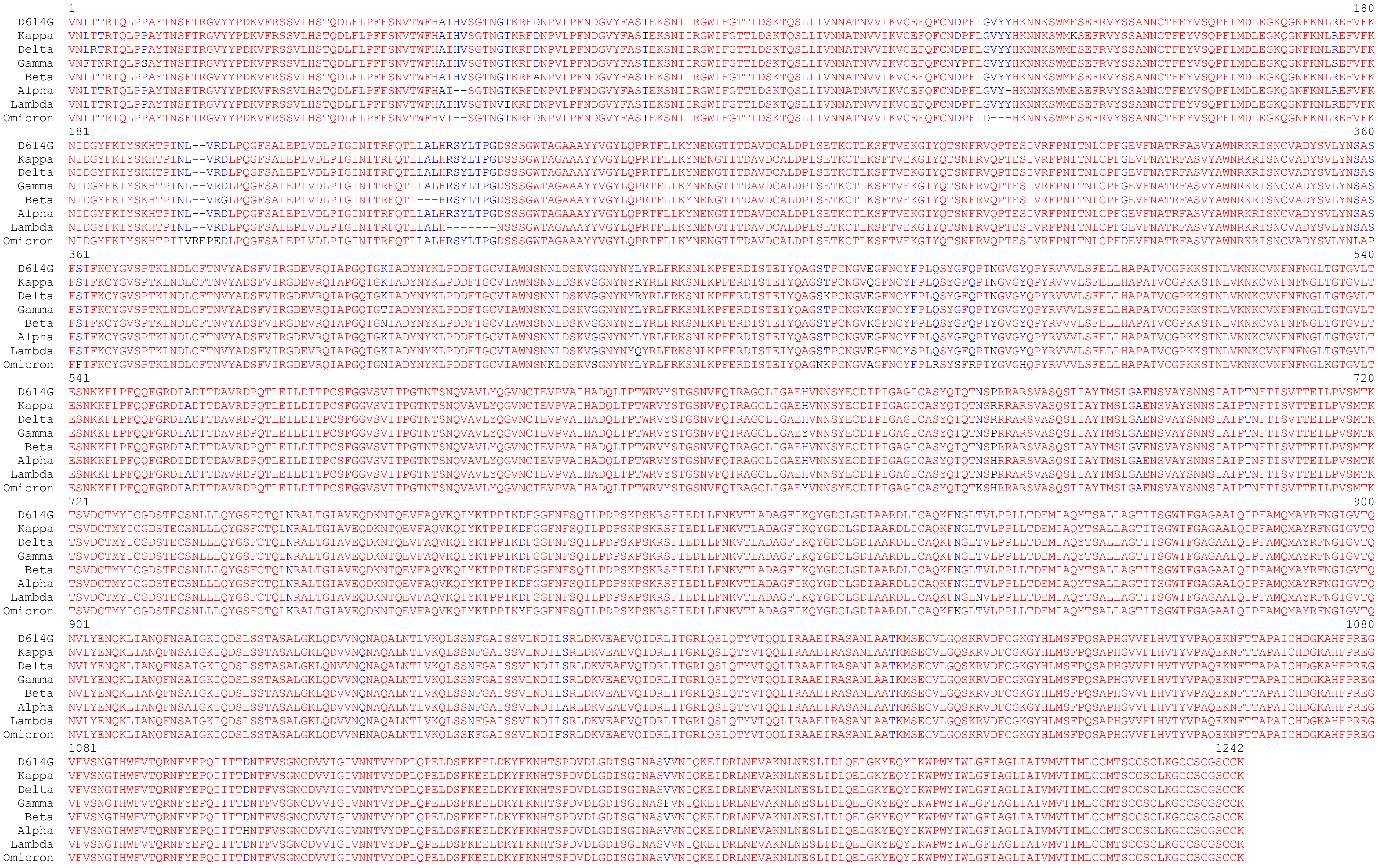

Fig. S11. SARS-CoV-2 S protein sequence alignment.

Input

	1	2	3	4	5	6	7	8	9	10	11	12
pCDNA-3.1-hNPC1-opt-3xFlag	+	+	+	+								
pCDNA-3.1-hNPC1-1-620-3xFlag					+	+	+	+				
pCDNA-3.1-hNPC1-1-377-3xFlag									+	+	+	+
pCDNA3.1-nCOV-S-wt-D19-HA	+				+				+			
pCDNA3.1-nCOV-S-D614G-D18		+				+				+		
pCDNA3.1-nCOV-S-Omicron-D18			+				+				+	
pCDNA3.1-nCOV-S-Delta-D18				+				+				+

IP

	1	2	3	4	5	6	7	8	9	10	11	12
	+	+	+	+								
					+	+	+	+				
									+	+	+	+
	+				+				+			
		+				+				+		
			+				+				+	
				+				+				+

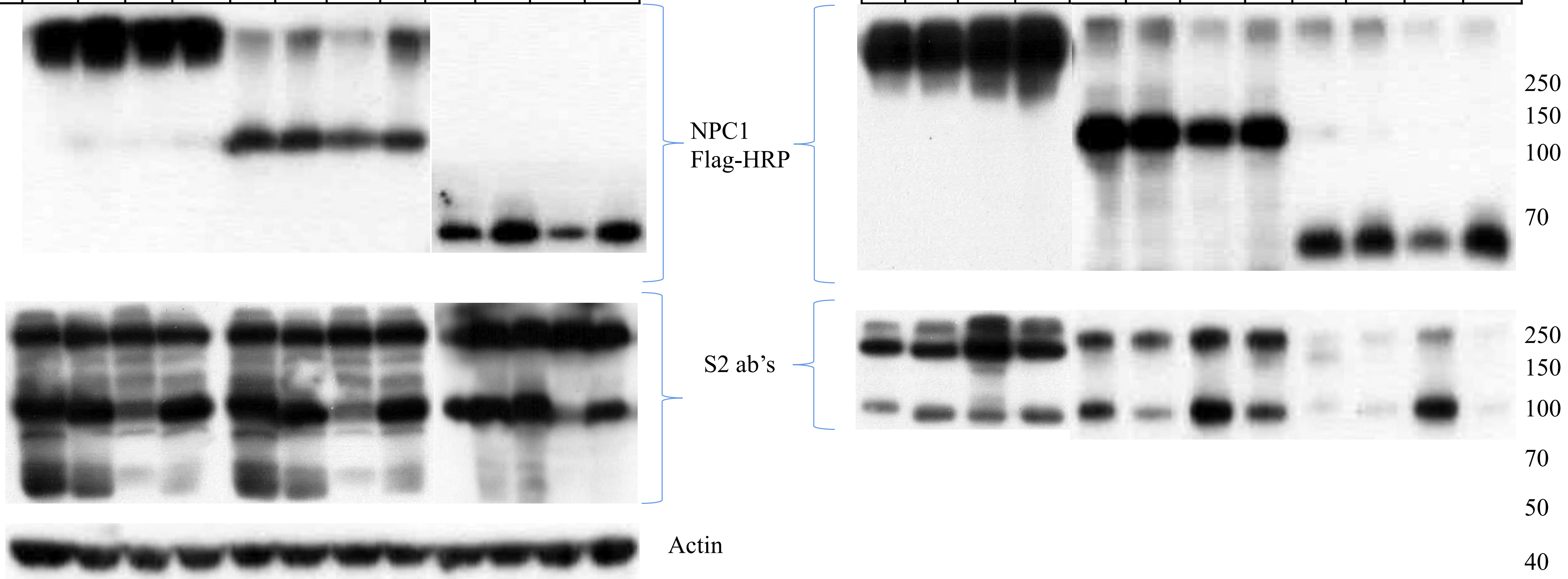

Fig. S12. NPC1 and its deletion mutants that only express its N-terminal 1-620 or 1-377 amino acids were expressed with S proteins from indicated SARS2 variants in HEK293T cells. These NPC1 proteins were immunoprecipitated and proteins were detected by western blots. Experiments were repeated 3 times independently, and representative results are shown.

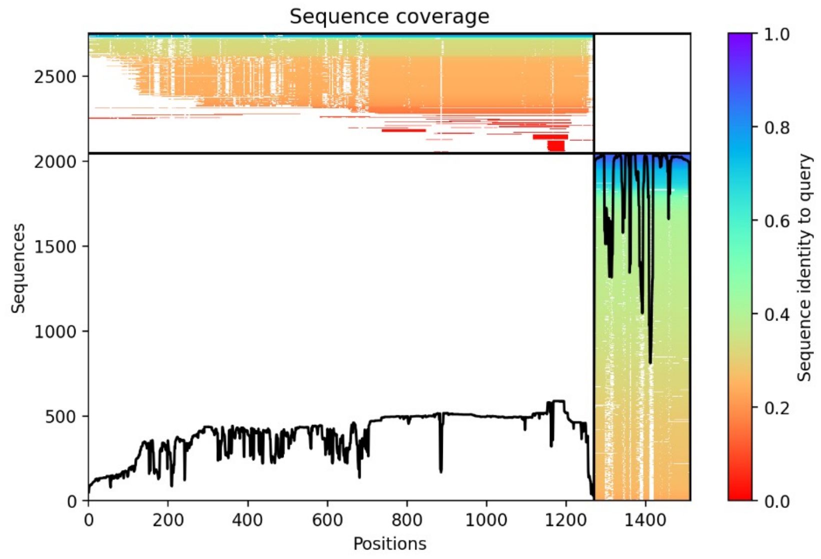

(a) Omicron/NPC1-C sequence coverage

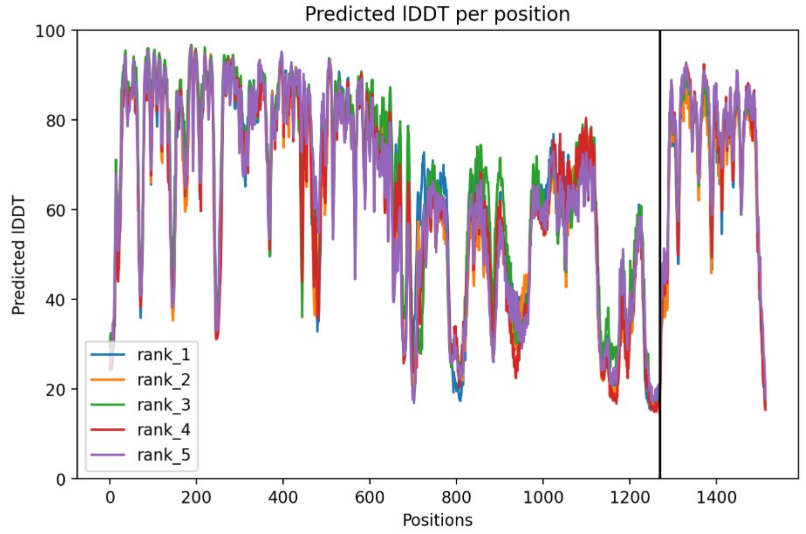

(b) Omicron/NPC1-C pLDDT score

Fig. S13. Omicron/NPC1-C complex sequence coverage and pLDDT. (a) The Omicron variant spans residues 1 to 1270, and the NPC1-C is represented by residues 1271 to 1514, totaling 244 residues. Both sequences exhibit a reliable multiple sequence alignment depth, surpassing 100. (b) The interacting region of Omicron (residue 340-510) and the entire structure of NPC1-C are predominantly in this range.

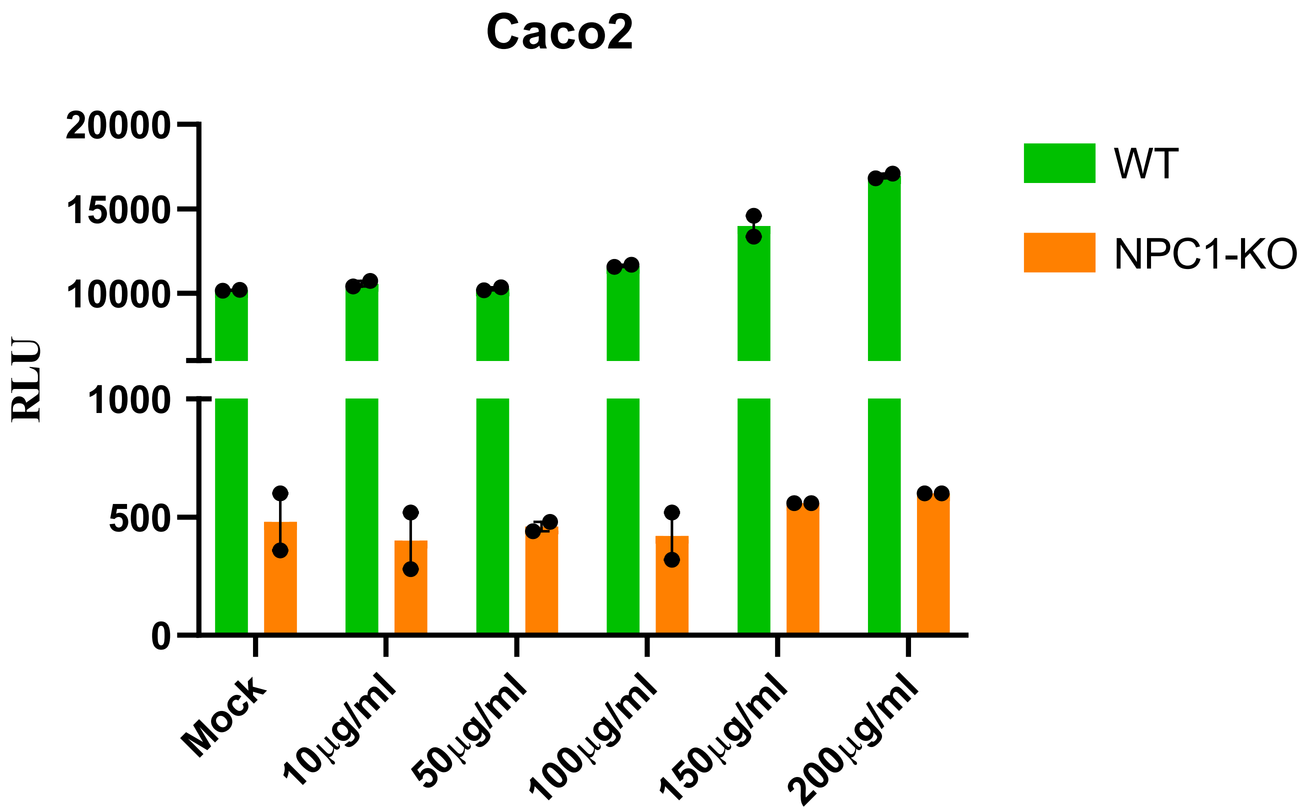

Fig. S14. Detection of the exogenous cholesterol activity in SARS2 infection. Indicated Caco2 cells were infected with SARS2-S pseudoviruses and treated with cholesterol at indicated concentrations. After 48 hours, viral infection was determined by measuring the intracellular luciferase activity. RLU, relative light unit.

Mapping target	Predicted binding interface on RBD
WT-S top-rank	375-S 376-T 398-D 400-F 401-V 402-I 403-R 404-G 405-D 406-E 407-V 408-R 409-Q 410-I 411-A 414-Q 415-T 416-G 417-K 418-I 419-A 420-D 421-Y 433-V 434-I 435-A 436-W 438-S 455-L 456-F 486-F 489-Y 493-Q 494-S 495-Y 496-G 497-F 498-Q 500-T 501-N 502-G 503-V 504-G 505-Y 506-Q 507-P 508-Y 509-R 510-V 511-V 512-V
WT-S most restricted consensus	375-S 376-T 400-F 401-V 402-I 403-R 404-G 405-D 406-E 407-V 408-R 409-Q 410-I 411-A 414-Q 415-T 416-G 417-K 418-I 433-V 435-A 436-W 438-S 495-Y 496-G 497-F 498-Q 500-T 501-N 502-G 503-V 504-G 505-Y 506-Q 507-P 508-Y 509-R 510-V
WT-S all possible	347-F 348-A 350-V 375-S 376-T 378-K 380-Y 398-D 399-S 400-F 401-V 402-I 403-R 404-G 405-D 406-E 407-V 408-R 409-Q 410-I 411-A 414-Q 415-T 416-G 417-K 418-I 419-A 420-D 421-Y 433-V 434-I 435-A 436-W 437-N 438-S 439-N 442-D 443-S 451-Y 455-L 456-F 486-F 489-Y 493-Q 494-S 495-Y 496-G 497-F 498-Q 499-P 500-T 501-N 502-G 503-V 504-G 505-Y 506-Q 507-P 508-Y 509-R 510-V 511-V 512-V
Omicron top-rank	346-S 372-F 398-V 399-I 400-R 401-G 402-D 403-E 404-V 405-R 414-N 415-I 434-N 436-N 439-D 440-S 441-K 442-V 443-S 444-G 445-N 446-Y 447-N 448-Y 449-L 450-Y 451-R 452-L 453-F 469-I 481-A 482-G 483-F 484-N 485-C 486-Y 487-F 488-P 489-L 490-R 491-S 492-Y 493-S 494-F 495-R 496-P 498-Y 499-G 500-V 501-G 502-H 503-Q 504-P 505-Y
Omicron most restricted consensus	400-R 402-D 453-F 490-R 498-Y 499-G 500-V 501-G 502-H
Omicron all possible	342-T 343-R 344-F 346-S 347-V 372-F 398-V 399-I 400-R 401-G 402-D 403-E 404-V 405-R 406-Q 407-I 408-A 410-G 411-Q 412-T 413-G 414-N 415-I 416-A 417-D 418-Y 419-N 420-Y 421-K 434-N 435-S 436-N 437-K 438-L 439-D 440-S 441-K 442-V 443-S 444-G 445-N 446-Y 447-N 448-Y 449-L 450-Y 451-R 452-L 453-F 454-R 455-K 456-S 457-N 458-L 459-K 460-P 469-I 470-Y 471-Q 472-A 473-G 474-N 481-A 482-G 483-F 484-N 485-C 486-Y 487-F 488-P 489-L 490-R 491-S 492-Y 493-S 494-F 495-R 496-P 497-T 498-Y 499-G 500-V 501-G 502-H 503-Q 504-P 505-Y 506-R

Table. S1. Binding interface of WT-S and Omicron

primer used for construction of plasmids		
**Plasmids**	**F**	**R**
pNL-ΔEnv-Luc	Han et al., 2008, Zhou et al., 2015, Wang et al., 2022, Yu et al., 2020, Zhang et al., 2022	
pNL-ΔEnv-GFP		
pNL-Env		
pcDNA3.1-VSV-G		
pcDNA3.1-EBOV-GP		
pcDNA3.1-EBOV-GPΔMLD		
pcDNA3.1-MARV-GP		
EBOV trVLPs		
pcDNA3.1-EBOV-GPΔMLD-HA	agacccaagctggctagcGCCACCATGTCTGCACTTCTGATCCTAGCTCTTGtTGGAGCTGCAGTTGCTATCC CACTTGGAGTCATCCA	ACGTCGTAGGGGTAACCGGTAAAGACAAATTTGCATATACA
pCAGGS-EBOV-VP40	ATCATTTTGGCAAAGAATTCGCCACCATGAGAAGAGTGATCCTGCCCA	GAACCTCCTCCACCGAATTCCTTCTCGATAACGGCAGGCAGG
pEGFP-N1-VP40	ATTCTGCAGTCGACGGTACCATGAGAAGAGTGATCCTGCCCA	TACCCATGGTGGCGACCGGTAACTTCTCGATCACGGCAGGCA
pCAGGS-SARS1-S-D19-FLAG	CCCAGACCAACTCCCCCtcaCGCGCCtcaTCCGTGGCCTCCCAGTCCA	aaaagatctgctagctcgagTCAGCAACAGCTGCCGCAGGAACA
pCAGGS-SARS2-S-D19-FLAG	ATCATTTTGGCAAAGAATTCGCCACCATGTTCGTGTTCCTGGTGCT	AAAAGATCTGCTAGCTCGAGTCAGCAGCAGGAGCCGCAGGAGCA
pCAGGS-MERS-S-D16-FLAG	catcattttggcaaagaattcGCCACCATGATCCACTCCGTGTTCCTGCT	AAAAGATCTGCTAGCTCGAGTCAGCAACATCTGTTGCACTTCAGCT
pcDNA3.1-NPC1-1-377-3FLAG	ggagacccaagctggctagcATGACAGCCAGGGGCCTGGCCCTGGGCCTGCTGCTGCTGCTCCTGTGCCCT GCCCAGGTGTTTAGCGTGACCACCAACCCTGTGGA	TCATCCTTGTAATCTCCGGAAGGGTTGGTGGTCACTCTCA
pcDNA3.1-NPC1-1-620-3FLAG	ggagacccaagctggctagcATGACAGCCAGGGGCCTGGCCCTGGGCCTGCTGCTGCTGCTCCTGTGCCCT GCCCAGGTGTTTAGCGTGACCACCAACCCTGTGGA	CATCCTTGTAATCTCCGGACACATCGCTGTCGGACTCCCTA
pcDNA3.1-NPC1-L656F-3FLAG	TGGACTCCAAGGTGAGCtTtGGCATCGCCGGCATCCT	ATCAGGATGCCGGCGATGCCaAaGCTCACCTTGGAGT
pcDNA3.1-NPC1-P691S-3FLAG	ATCGTGATCGAGGTCATTtCCTTCCTGGTGCTGGC	ACGGCCAGCACCAGGAAGGaAATGACCTCGATCAC
pcDNA3.1-NPC1-D786N-3FLAG	GTGAGCCTGCTGGGCCTGaATATCAAGAGGCAGGA	TTCTCCTGCCTCTTGATATtCAGGCCCAGCAGGCT
pLenti-BSD-hACE2	atgtcaagctcttcctggctccttc	ctaaaaggaggtctgaacatcatcag
pLenti-BSD-hTMPRSS2	ATGGCGCTGAATTCCGGATCA	TTAGCCATCGGCACGCATTTGTCTG

Table. S2. List of primers used for construction of plasmids

**Viruses**	**Target**	**(5’ - 3')**
Influenza virus	viral RNAs	AGCRAAAGCAGG
	amplify NP gene	TGCTCACAAGTCCTGCCTGC
		GGCATGCCATCCACACCAGT
	amply GAPDH	TCAAGAAGGTGGTGAAGCAG
		GAGGGGAGATTCAGTGTGGT
Authentic SARS-CoV-2	viral N gene	GGGGAACTTCTCCTGCTAGAAT
		CAGACATTTTGCTCTCAAGCTG

Table. S3. List of primers used for virus-specific gene amplification

**Target cells**	**sgRNA's**	**5'-3'**	**3'-5'**
gRNA target human NPC1	Oligo1	GGCCCCCTCAAGTAATGACA	TGTCATTACTTGAGGGGGCC
	Oligo2	TCTGTTAATGCAAGTGACAA	TTGTCACTTGCATTAACAGA
	Oligo3	TTCAAAAACTGACTCTGTCG	CGACAGAGTCAGTTTTTGAA
	Oligo4	GGGTACATCAGCTCCCGAA	TTCGGGAGCTGATGTACCC
	Oligo5	TCGTGTTATACGGTGAAAG	CTTTCACCGTATAACACGA
gRNA target Chlorocebus sabaeus NPC1	Oligo1	TGAGTAGTAGCTAGGACTAC	GTAGTCCTAGCTACTACTCA
	Oligo2	TCTTGCTCTGTTGCCCAGGC	GCCTGGGCAACAGAGCAAGA
gRNA target hamster npc1	Oligo1	CACCAACTCCCGTGCCCTGG	CCAGGGCACGGGAGTTGGTG
	Oligo2	ATCTATGAGCCGTACCCCTC	GGAGGGGTACGGCTCATAGAT
siRNA for human NPC1	siRNA #1	CAGAuuACCuGuuuCGuGAtt	
	siRNA #2	CCCuCGACAGAGuCAGuuutt	
	negative control (NC) siRNA	UUCUCCGAACGUGUCACGUTT	

Table. S4. List of small guide (sg) RNAs that target *NPC1* genes in human cells, Vero-E6 cells, and CHO cells
