# Supplementary material for: Tubeimosides are pan-coronavirus and filovirus inhibitors that can block their fusion protein binding to Niemann-Pick C1
Source: Nat Commun. 2024 Jan 2;15:162. doi: 10.1038/s41467-023-44504-4 (PMC10762260; doi:10.1038/s41467-023-44504-4)
Supplement: Supplementary file 3 — Reporting Summary [file 41467_2023_44504_MOESM3_ESM.pdf]

nature portfolio

Corresponding author(s): Zheng, Yong-Hui

Last updated by author(s): Dec 6, 2023

Reporting Summary

Nature Portfolio wishes to improve the reproducibility of the work that we publish. This form provides structure for consistency and transparency in reporting. For further information on Nature Portfolio policies, see our Editorial Policies and the Editorial Policy Checklist.

Statistics

For all statistical analyses, confirm that the following items are present in the figure legend, table legend, main text, or Methods section.

n/a	Confirmed	
☐	☒	The exact sample size (*n*) for each experimental group/condition, given as a discrete number and unit of measurement
☐	☒	A statement on whether measurements were taken from distinct samples or whether the same sample was measured repeatedly
☐	☒	The statistical test(s) used AND whether they are one- or two-sided<br>*Only common tests should be described solely by name; describe more complex techniques in the Methods section.*
☒	☐	A description of all covariates tested
☒	☐	A description of any assumptions or corrections, such as tests of normality and adjustment for multiple comparisons
☐	☒	A full description of the statistical parameters including central tendency (e.g. means) or other basic estimates (e.g. regression coefficient) AND variation (e.g. standard deviation) or associated estimates of uncertainty (e.g. confidence intervals)
☐	☒	For null hypothesis testing, the test statistic (e.g. *F*, *t*, *r*) with confidence intervals, effect sizes, degrees of freedom and *P* value noted<br>*Give P values as exact values whenever suitable.*
☒	☐	For Bayesian analysis, information on the choice of priors and Markov chain Monte Carlo settings
☒	☐	For hierarchical and complex designs, identification of the appropriate level for tests and full reporting of outcomes
☒	☐	Estimates of effect sizes (e.g. Cohen's *d*, Pearson's *r*), indicating how they were calculated

Our web collection on statistics for biologists contains articles on many of the points above.

Software and code

Policy information about availability of computer code

Data collection	No software was used for data collection.
Data analysis	GraphPad Prism (Version 9.3.1) was used for the data analysis. Adobe Photoshop 2022 was used to analyze western blot images. Adobe Illustrator 2022 was used to create figures. SnapGene 6.0.2 was used to create vector maps. BioRender was used for making the model (https://biorender.com). qPCR results were analyzed using Bio-Rad CFX Manager 3.1. Western blots were quantified with ImageJ Launcher 1.4.3.67 (https://imagej.nih.gov/ij/). Confocal images were analyzed using ZEISS Zen Software version 2.1. Chemical structures were drawn by ChemDraw 22.2.0.

For manuscripts utilizing custom algorithms or software that are central to the research but not yet described in published literature, software must be made available to editors and reviewers. We strongly encourage code deposition in a community repository (e.g. GitHub). See the Nature Portfolio guidelines for submitting code & software for further information.

Data

Policy information about availability of data

All manuscripts must include a data availability statement. This statement should provide the following information, where applicable:

- Accession codes, unique identifiers, or web links for publicly available datasets
- A description of any restrictions on data availability
- For clinical datasets or third party data, please ensure that the statement adheres to our policy

A source data file is provided with this paper.

Research involving human participants, their data, or biological material

Policy information about studies with human participants or human data. See also policy information about sex, gender (identity/presentation), and sexual orientation and race, ethnicity and racism.

Reporting on sex and gender	N/A
Reporting on race, ethnicity, or other socially relevant groupings	N/A
Population characteristics	N/A
Recruitment	N/A
Ethics oversight	N/A

Note that full information on the approval of the study protocol must also be provided in the manuscript.

Field-specific reporting

Please select the one below that is the best fit for your research. If you are not sure, read the appropriate sections before making your selection.

☒ Life sciences ☐ Behavioural & social sciences ☐ Ecological, evolutionary & environmental sciences

For a reference copy of the document with all sections, see nature.com/documents/nr-reporting-summary-flat.pdf

Life sciences study design

All studies must disclose on these points even when the disclosure is negative.

Sample size	Sample size was determined based on our routine experiments in cell biology and virology. At each point of data collection, at least two samples were collected for data analysis. No significant variation was observed, indicating the sample size was sufficient.
Data exclusions	No data were excluded.
Replication	Experiments were repeated at least two or three times. All attempts at replication were successful.
Randomization	Proper controls were included in each experiments. No randomization design was necessary.
Blinding	Proper controls were included in each experiments. No blinding design was necessary.

Reporting for specific materials, systems and methods

We require information from authors about some types of materials, experimental systems and methods used in many studies. Here, indicate whether each material, system or method listed is relevant to your study. If you are not sure if a list item applies to your research, read the appropriate section before selecting a response.

Materials & experimental systems

n/a	Involved in the study
☐	☒ Antibodies
☐	☒ Eukaryotic cell lines
☒	☐ Palaeontology and archaeology
☒	☐ Animals and other organisms
☒	☐ Clinical data
☒	☐ Dual use research of concern
☒	☐ Plants

Methods

n/a	Involved in the study
☒	☐ ChIP-seq
☐	☒ Flow cytometry
☒	☐ MRI-based neuroimaging

Antibodies

Antibodies used	Goat anti-human ACE2 affinity purified IgG (Cat No. AF933) was from R&D Systems;<br>rabbit anti-human NPC1 (Cat No. ab134113) was from Abcam;<br>rabbit anti-SARS-CoV-spike protein (Cat No. NB100-56047) was from Novus Biologicals;<br>rabbit anti-SARS-CoV-2 spike RBD protein (Cat No. 40592-T62), rabbit anti-Zaire EBOV-GP (Cat No. 40442-T48) were from Sino Biological;<br>mouse monoclonal anti-VSV-G (Cat No. Cat#A02180) was from Abbkine;<br>rat monoclonal anti-FLAG (Cat No. F3165), horseradish Peroxidase (HRP)-conjugated anti-FLAG (Cat No. A8592), HRP-conjugated anti-HA (Cat No. H6533), and HRP-conjugated anti-β-Actin (Cat No. A3854) were from Sigma Aldrich;<br>HRP-conjugated AffiniPure donkey anti-goat IgG (H+L) (Cat. No. 705-035-003), HRP-conjugated goat anti-mouse IgG (Cat No. 115-035-003) and anti-rabbit IgG (Cat No. 111-035-003), and APC-conjugated polyclonal anti-goat antibody (Cat No. 705-136-147) were from Jackson ImmunoResearch.
Validation	All commercial antibodies used in this study were validated by the manufactures. Data sheets for these commercial antibodies are provided on the manufacturers' websites with antibody profiles and citations.

Eukaryotic cell lines

Policy information about cell lines and Sex and Gender in Research

Cell line source(s)	HEK293T (CRL-3216), Caco-2 (HTB-37), Calu-3 (HTB-55), Vero-E6 (CRL-1586), SNB-19 (CRL-2219), A549 (CCL-185), and CHO-K1 (CCL-61) cell lines were purchased from American Type Culture Collection (ATCC). TZM-bl cell line (ARP-8129) was obtained from NIH HIV Reagent Program. Huh-7 cell line (1101HUM-PUMC000679) was purchased from Institute of Basic Medical Sciences (IBMS), Chinese Academy of Medical Sciences (CAMS) & Peking Union Medical College (PUMC).
Authentication	Because all these cell lines have clear track-records and commonly used, they were not recently authenticated.
Mycoplasma contamination	All these cell lines were negative for mycoplasma contamination.
Commonly misidentified lines<br>(See ICLAC register)	No commonly misidentified cell lines were used.

Flow Cytometry

Plots

Confirm that:

☒ The axis labels state the marker and fluorochrome used (e.g. CD4-FITC).

☒ The axis scales are clearly visible. Include numbers along axes only for bottom left plot of group (a 'group' is an analysis of identical markers).

☒ All plots are contour plots with outliers or pseudocolor plots.

☐ A numerical value for number of cells or percentage (with statistics) is provided.

Methodology

Sample preparation	A549 and A549-ACE2 cells were stained with the primary anti-ACE2 antibody (R&D, cat: AF933) and then secondary APC-conjugated anti-goat antibody (Jackson, Cat No. 705-136-147). After being washed twice with PBS, stained cells were analyzed by flow cytometry.
Instrument	LSR II (BD)
Software	BD FACSDiva v7.0; FlowJo v10.8.
Cell population abundance	The ACE2-positive cell populations were over 60% in A549-ACE2 cells.

Gating strategy	Forward versus side scatter (FSC vs SSC) gating was used to identify cells of interest based on size and granularity (complexity).

☐ Tick this box to confirm that a figure exemplifying the gating strategy is provided in the Supplementary Information.
